# Strategies for Antimicrobial Peptides Immobilization on Surfaces to Prevent Biofilm Growth on Biomedical Devices

**DOI:** 10.3390/antibiotics11010013

**Published:** 2021-12-23

**Authors:** Mathieu Nicolas, Bruno Beito, Marta Oliveira, Maria Tudela Martins, Bruno Gallas, Michèle Salmain, Souhir Boujday, Vincent Humblot

**Affiliations:** 1 Sorbonne Université, UMR 7197, Laboratoire de Réactivité de Surface, Centre National de la Recherche Scientifique (CNRS), 4 Place Jussieu, F-75005 Paris, France; mathieu.nicolas@insp.jussieu.fr; 2 Sorbonne Université, Institute of Nanosciences Paris (INSP), Centre National de la Recherche Scientifique (CNRS), 4 Place Jussieu, F-75005 Paris, France; gallas@insp.jussieu.fr; 3 Sorbonne Université, Master de Chimie, Profil MatNanoBio, Faculté des Sciences et Ingénierie of Sorbonne Université, 4 Place Jussieu, F-75005 Paris, France; beitobruno_17@hotmail.com (B.B.); Marta.rdo.97@gmail.com (M.O.); maria.tudela.martins@gmail.com (M.T.M.); 4 Sorbonne Université, Institut Parisien de Chimie Moléculaire (IPCM), Centre National de la Recherche Scientifique (CNRS), 4 Place Jussieu, F-75005 Paris, France; michele.salmain@sorbonne-universite.fr; 5 Franche-Comté Électronique Mécanique Thermique et Optique-Sciences et Technologies (FEMTO-ST) Institute, Centre National de la Recherche Scientifique (CNRS), UMR 6174, Université Bourgogne Franche-Comté, 15B Avenue des Montboucons, F-25030 Besançon, France

**Keywords:** biofilms, AMP, antimicrobial, peptide, immobilization, biofunctionalization

## Abstract

Nosocomial and medical device-induced biofilm infections affect millions of lives and urgently require innovative preventive approaches. These pathologies have led to the development of numerous antimicrobial strategies, an emergent topic involving both natural and synthetic routes, among which some are currently under testing for clinical approval and use. Antimicrobial peptides (AMPs) are ideal candidates for this fight. Therefore, the strategies involving surface functionalization with AMPs to prevent bacterial attachment/biofilms formation have experienced a tremendous development over the last decade. In this review, we describe the different mechanisms of action by which AMPs prevent bacterial adhesion and/or biofilm formation to better address their potential as anti-infective agents. We additionally analyze AMP immobilization techniques on a variety of materials, with a focus on biomedical applications. Furthermore, we summarize the advances made to date regarding the immobilization strategies of AMPs on various surfaces and their ability to prevent the adhesion of various microorganisms. Progress toward the clinical approval of AMPs in antibiotherapy is also reviewed.

## 1. Introduction

Biofilms infections caused by implants and medical devices through nosocomial diseases are increasingly present in hospitals, and in order to avoid patient’s lives being impacted, novel approaches are needed to prevent these infections. These pathologies have led to the development of numerous antifouling strategies, an emergent topic involving both natural and synthetic routes, among which some are currently under testing for clinical approval and use. By definition, antifouling is the property by which specially designed coatings prevent biofouling. Antifouling can be achieved by preventing bacterial adhesion to surfaces (anti-adhesive strategy) or by exerting a biocidal action (bactericidal or bacteriostatic strategies). Antimicrobial peptides (AMPs) are ideal candidates for the latter. Therefore, the strategies involving surface functionalization with AMPs have experienced a tremendous development over the last decade to prevent bacterial attachment by exerting a biocidal action. In this review, we provide an overview of the methods of immobilization of AMPs on a variety of materials, with a focus on biomedical applications. We additionally analyze the different mechanisms of action by which AMPs prevent biofilm formation to better understand their potential as anti-infective agents.

It becomes clear that, with their reliable mechanisms of protection and communication, biofilms are not only extremely hard and costly to remove, but also very resistant to current antibiotic treatments [1]. Thus, a new preventive approach has been taken to address this issue, involving antimicrobial peptides as a viable solution. These peptides, which are part of the immune response of several organisms against microbial invasion, can be immobilized onto surfaces to prevent the formation of biofilms at their early stages so that removal of the unwanted microorganisms in a later stage becomes unnecessary [2]. Currently, more research is being put into this subject and, along with AMP coatings, numerous techniques for preventing biofilms formation are being investigated [3,4,5] such as interception of the quorum sensing (QS) system [6], microstructure topographic exploitation [7], use of alternative antibacterial compounds like quaternary ammonium salts [8], among others.

Conceptually, AMPs represent a promising alternative to antibiotics when it comes to overcoming bacterial resistance to treatment. However, their clinical approval is still pending due to insufficient efficiency and possible toxicity to the surrounding tissues. In what follows, we will focus on the biomedical applications of AMPs and discuss their potential as therapeutic agents to fight biofilm infections. We will describe different AMPs and their utility, along with the diversity of surfaces where they can be immobilized.

## 2. Biofilms Formation and Resistance

Biofilms, although yet to be named at that time, were first scientifically referenced by Anton van Leeuwenhoek in the 17th century by his description of “*animaculi*” in his own dental plaque. Although it took a long time for this observation to be recognized by his pairs, he is now considered as a pioneer in the field of microbiology [9,10].

**Figure 1:**
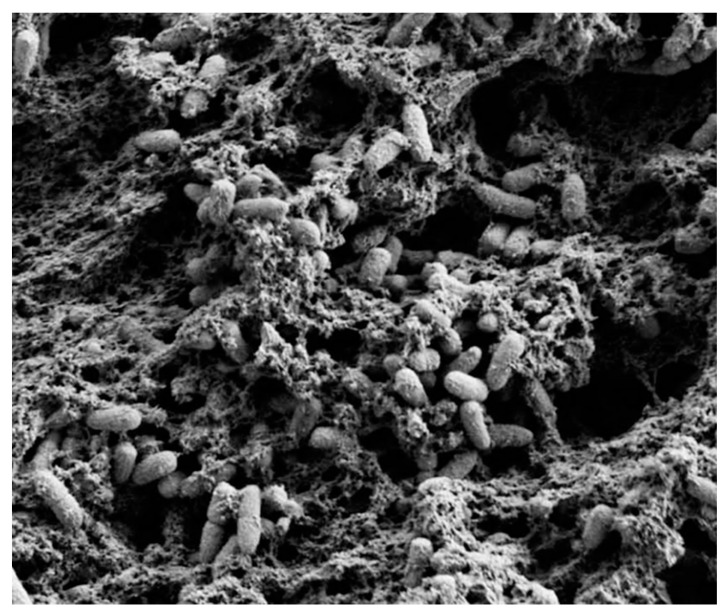
Scanning electron microscopy image of *P. aeruginosa* PA14, cultured as a pellicle. Reproduced from [24]. Copyright © 2011 Franklin et al.

Currently, biofilms are defined as an irreversibly attached microbial aggregates incorporated in a matrix of self-produced extracellular polymeric substances (EPSs), Figure 1. This matrix is composed of polysaccharides, extracellular DNA and various proteins, glycoproteins and glycolipids [11,12,13]. In advantage to planktonic bacterial cells, these sessile communities thrive in a range of different environments and hosts due to their phenotypically distinctive mode of growth [14,15]. Along with this protective mechanism, biofilms comprise a circulatory system by which the colonies can receive nutrients. In addition, biofilms also hold the ability to coordinate gene expression conforming to cell density, phenomenon known as quorum sensing [16,17].

These features make biofilms highly infectious and enhance their resistance to treatment. In fact, these microorganisms are responsible for past and present large epidemic infections. They still affect millions of individuals by causing microbial infections [18], especially through implanted medical devices [19,20]. Furthermore, biofilms are also a cause for concern in a very significant number of industrial fields, such as food, paper, air conditioning, and water processing, to name a few [21,22,23]. For all of these reasons, the hazards of biofilm formation and the urgency to find more efficient and effective ways to eliminate them are topics of ongoing investigations, due in part to the great source of expenditure in money and resources that it represents in the present day.

One major issue when dealing with biofilms, from a biomedical point of view, is their intrinsic resistance to antimicrobial agents. While planktonic bacterial cells can be eliminated most of the time with proper antibiotics, bacteria in their sessile state (i.e., mature irreversible biofilms) show, in most cases, a much lower vulnerability to antimicrobial treatment [15,25]. This phenomenon can be explained by multiple factors among which the fact that the EPSs matrix prevents the antimicrobial agents from reaching deep into the biofilm and therefore defies the diffusion of antibiotics. Another reason for biofilms resistance is the fact that when there is a nutrient limitation (as it could be the case for a mature biofilm), bacteria slow their growth rate and behave as if in a stationary phase. Therefore, it is believed that stationary phase can be responsible for the insusceptibility of biofilms to antibiotics. Amongst several others, these defense strategies provide biofilms with a powerful protective response to antibacterial treatment [2].

**Figure 2:**
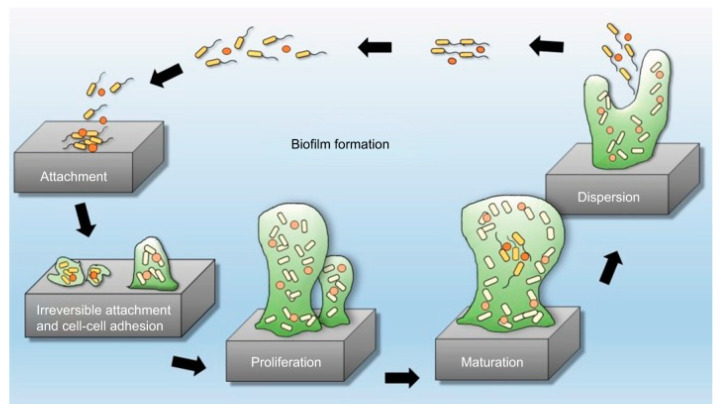
Five stages of biofilm development. Reproduced from [29]. Copyright © 2018 Abu Bakar et al. Reproduced by permission from Perfectus Biomed Limited. http://perfectusbiomed.com/cbe-meeting-anti-biofilm-technologies/.

The high resistance of biofilms to antimicrobial agents is partly understandable when investigating the mechanism of their formation. The complex process of biofilm formation starts with an initial attachment of planktonic cells onto the surface, Figure 2. In the particular case of the surface of biomedical devices, this adhesion process can sometimes be facilitated by proteins and glycoproteins that coat the surface as a natural reaction of the host to the device [26]. The rate of attachment is also dependent on the characteristics of the microbial cells, such as the presence of appendages [27]. The reversible attachment phase is followed by progression to a sessile state, where the quorum sensing system is initiated within the microcolonies and when the building process of the biofilm becomes irreversible, Figure 2. The cells secrete the polysaccharides that make up the matrix of EPSs, which gives structure to the now maturated biofilm. After maturation, cells begin to detach from the biofilm, initiating the dispersion phase by induction of disassembly factors [28].

To conclude, owing to their reliable mechanisms of protection and communication, biofilms are therefore extremely hard and costly to remove, but also very resistant to current antibiotic treatments. In what follows we will focus on the biomimetic approach relying on the use antimicrobial peptides (AMPs).

## 3. Antimicrobial Peptides (AMPs) Structure and Mechanism of Action

AMPs are short amphiphilic peptides composed of 12 to 100 amino acids, present in the first line of defense of multiple organisms [30,31,32]. They were first isolated in the 1980s from the frog species *Xenopus laevis*, whose skin provides a defense mechanism against microbes through the presence of one particular cationic AMP: magainin, an amphipathic α-helix 23 AA-residues peptide [33]. This AMP prevents formation of biofilm by disrupting the bacterial cell membrane [34], without interfering with the host cells.

To date, the antibacterial polymer database (APD, http://aps.unmc.edu/AP/) lists 3273 AMPs among which 369 bacteriocins (from bacteria), 5 AMPs from archaea, 8 from protists, 22 from fungi, 361 from plants, and 2424 from animals, the main source of AMPs. These peptides have among others antibiofilm, antiprotozoal action [2,35], antibacterial [36], antiviral [37], antifungal [38], antiparasitic [39], anticancer [40], antioxidant [41], chemotactic [42], insecticidal [36], or even wound healing [43] properties.

**Figure 3:**
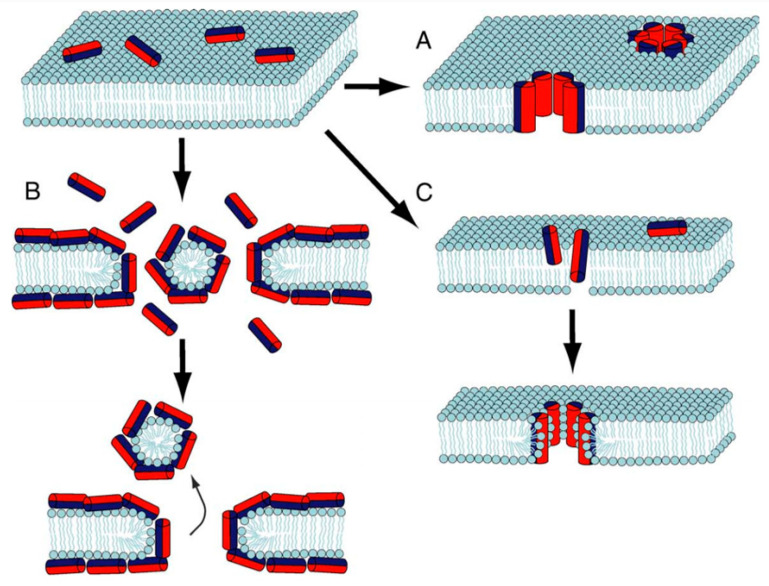
Antimicrobial peptide mechanisms of action. (**A**). The barrel-stave model. (**B**) The carpet model. (**C**) The toroidal or wormhole pore model. Adapted from [48]. Copyright © 2012 Elsevier Ltd. All rights reserved.

The mechanism of action of AMPs responsible for their antibacterial activity is based on the integration and consecutive disruption of the phospholipid bilayer of microbial cells, which can be achieved by different pathways. The main models are the barrel-stave, the toroidal (or wormhole) pore and the carpet models, as shown in Figure 3. In the barrel-stave mechanism, the peptides penetrate the membrane and form a pore so that the hydrophobic regions of the peptides face the lipid core, and the hydrophilic regions outline the inside of the pore, as shown in Figure 3A. The carpet model is a result of the parallel orientation of the peptides with respect to the lipid bilayer surface and formation of a peptide carpet, disrupting the membrane, as shown in Figure 3B. Finally, in the toroidal pore model (Figure 3C), the peptides induce the phospholipid layer molecules to curve inwards, creating a pore where the hydrophilic regions of the peptides and the lipid groups of the layer molecules face the interior of the pore [44,45,46,47]. In addition to these three main models, several other mechanisms also characterize the mode of action of certain AMPs, including strategies that diverge from a membrane-disruption approach [46].

**Figure 4:**
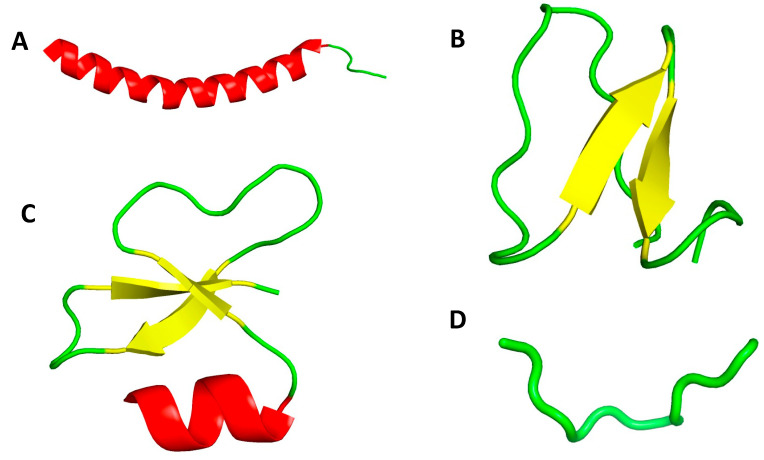
Three-dimensional (3D) structures of representative antimicrobial peptides. (**A**) α-helical Scheme 37. (PDB code: 2K6O); (**B**) β-sheet structure of plant kalata B1 (PDB code: 1NB1); (**C**) αβ structure of human β-defensin-1 HBD-1 (PDB code: 1IJV); (**D**) non-αβ structure of cattle indolicidin (PDB code: 1G89).

Therefore, antimicrobial peptides can be categorized according to different features. One major feature is their secondary and tertiary structures that differ from aqueous solvents to a membranomimetic medium, as well as their interactions with membrane vesicles. Based on these three-dimensional secondary and tertiary structures, which can be determined by NMR spectroscopy [49], AMPs are divided into four categories as depicted in Figure 4: (i) α when the major secondary structure of the peptide is α-helical, (ii) β if the structure contains at least a pair of two-strands, (iii) αβ for peptides with both types of structure, (iv) non-αβ if none of the structures are present. The secondary and tertiary structures of AMPs are believed to play an important role in the mechanism of action against microbial cells, particularly in the process of integration in the cell membrane [47], albeit in 2021 only about 40% of AMPs have in fact a known 3D structure [50].

Another classification of AMPs divides them into four different families, which additionally includes the amino acid composition. This categorization includes cationic AMPs, which can be (i) linear helical peptides (that is to say non-cyclic peptides with a helical secondary structure), (ii) peptides enriched with one amino acid, (iii) peptides with one disulfide bridge and (iv) peptides with more than one disulfide bridge [44].

One major advantage of antimicrobial peptides that focused attention as a possible alternative to conventional antibiotic treatments is the low development of resistance by these antimicrobial agents, which overcomes the crucial problem of dangerously rapid ineffectiveness of regular therapies when combating sensitive bacterial strains [51,52]. Furthermore, AMPs also show activity against a broad range of microorganisms. The natural defense system via antimicrobial peptides present in a wide variety of animal, insect and plant species suggested the use of AMPs as therapeutic agents against biofilm infections, either on their own or incorporated in a classic antibiotic treatment for a synergistic enhancement of effectiveness [53,54].

Despite their great potential as anti-infective agents, clinical approval of AMPs has been setback for various reasons. In 2019, Koo and coworkers [55] reported that 70 natural and synthetic AMP were in preclinical and clinical trials at different stages with only 2% being FDA approved to date. Systemic and local toxicity, questionable proteolytic stability, pH sensitivity and production cost are among the critical drawbacks that have prevented FDA approval of AMPs as therapeutic agents. In other cases, treatment with antimicrobial peptides has shown no obvious advantages over conventional antibiotics [53,56,57,58]. A more detailed list of the antimicrobial peptides that have undergone clinical and preclinical trials is documented in Table 1.

It has become clear over the last 2 decades, that the immobilization techniques for surfaces coating represent a crucial factor in the success of AMPs for preventing biofilm formation or bacterial contamination. In the next section, we intent to summarize the work developed to date with an in-depth description of immobilization strategies for a wide range of surfaces.

## 4. Strategies for AMP Immobilization on Surfaces

It is widely accepted that the ideal strategy for successful anti-fouling activity can be achieved by preventing initial attachment of microbial agents, either by anti-adhesive or bactericidal/bacteriostatic approaches. The immobilization techniques for binding AMPs onto surfaces have proven to be extremely relevant to determine levels of performance [51,63]. Furthermore, a successful immobilization is crucial to avoid or control AMP diffusion and limit toxicity of the antimicrobial coating.

Immobilization can be achieved by a number of different strategies, either by physical methods (adsorption, Layer-by-Layer (LbL)) or chemical methods (covalent bonding, Self-Assembled Monolayers (SAMs)). Recent developments tend to focus on covalent immobilization approaches for the coating of surfaces due to increased stability and efficacy of antimicrobial activity, partnered with decrease in both toxicity levels and AMP diffusion [64,65,66]. Another important factor to consider when performing the immobilization of AMPs on surfaces lies in the low surface concentrations needed for high antimicrobial efficacy.

A broad range of solid surfaces has been modified by numerous antimicrobial peptides, including polymers, titanium, gold, stainless steel and glass, some of which are addressed in more detail in the following review [65]. Regardless of surface type, several key factors strongly influence antimicrobial performance of AMPs and should be considered for successful immobilization and peptide activity. These factors range from coverage or surface density as mentioned previously [67], but also orientation, distance to the surface [68] or even lateral mobility [69]. An overview on AMP immobilization methods is given in Table 2, detailing the strategies utilized, along with the type of bound AMP, solid support, and invading microorganism.

We mainly focus herein on nosocomial diseases and medical device-related biofilm infections. Functionalized AMPs for a direct treatment of an infection are off topic, even if interesting studies have been carried out—for instance a very recent study published in 2021 [70] about hydrogels functionalized with peptide RRP9W4N thanks to EDC-NHS chemistry that exhibit strong antibacterial activity. Research on resins, not related to the biomedical field, are not reviewed here but also deserve to be mentioned such as the works of Bagheri et al. [68], about peptides linked to resins thanks to PEG spacers where the influence of the spacer’s length on the antibacterial activity was studied; in that particular case, the decrease of activity due to spacer length variation cannot be compensated by an increase in surface density of the peptide.

**Table 2:** Overview of AMP immobilization on solid surfaces and their antimicrobial activity.

AMP	Substratum	Immobilization Strategy	Studied Microorganisms	Reference
Magainin I	Gold surface	Self-assembled monolayer	*L. ivanovii*, *E. faecalis*, *S. aureus*	Humblot et al., (2009) [71]
LL37	Titanium surface	Site-specific conjugation via amine-reactive NHS and thiol-reactive maleimide moieties	*E. coli*	Gabriel et al., (2006) [72]
HHC36	Titanium surface	CuAAC click chemistry: Titanium silanized with APTS and peptide modified with PEG	*S. aureus*, *E. coli*	Chen et al., (2019) [73]
Tet-213	Titanium slide	Tethering on copolymer brushes of N,N-dimethylacrylamide/N-(3-aminopropyl)-methacrylamide hydrochloride	*P. aeruginosa*	Gao et al., (2011) [74]
Tet-213, 1010cys, Tet-20, Tet-21, Tet-26, HH2, MXX226	Titanium slide	Covalently grafted hydrophilic polymer brushes conjugated with peptides	*S. aureus*, *P. aeruginosa*	Gao et al., (2011) [75]
Magainin I	Silicon wafer	Tethering on copolymer brushes of 2-(2 methoxyethoxy) ethylmethacrylate/hydroxyl-terminated oligo (ethylene glycol) Poly(MOE2MA-co-HOEGMA)	*L. ivanovii*, *B. cereus*	Glinel et al., (2009) [76]
CW11	Polydimethylsiloxane (PDMS)	Cross-linking of peptides to allylglycidyl ether modified PDMS surface (PDMS-AGE-PEG) via Sulfhydryl Chemistry	*E. Coli*, *S. aureus*, *P. aeruginosa*	Lim et al., (2013) [77]
RK1 and RK2	Silicone urinary catheter and Polydimethylsiloxane (PDMS)	Cross-linking of peptides to allylglycidyl ether modified PDMS surface (PDMS-AGE)	*E. Coli*, *S. aureus*, *C. albicans*	Li et al., (2014) [78]
Temporin-SHa	Titanium surface	Covalent immobilization on Si-O-Ti groups	*S. epidermidis*, *E. coli*	Masurier et al., (2018) [79]
122 variant peptides of 2 starting sequences: Bac2A and Indolicidin	Cellulose support	Cellulose-amino-hydroxypropyl ether (CAPE) linker chemistry; or directly synthetized onto a bifunctional resin; or directly bound to the microtiter plate via biotin-streptavidin interaction	*S. aureus*, *P. aeruginosa*, *C. albicans*	Hilpert et al., (2009) [80]
TBKKG6A and lasioglossin-III	Cellulose support	cysteine-cellulose conjugate coupled to obtaining thioester peptides either on the C-terminal or the N-terminal part of the molecules	*E. coli.*	Sperandeo et al., (2020) [81]
Antibacterial hybrid peptide	Silicone catheter	Covalent immobilization on silanol groups	*E. Coli*, *P. aeruginosa*, *S. aureus*	Pinese et al., (2016) [82]
Bioactive peptides	Silicone dressing	Covalent immobilization on silanol groups	Studies of peptides bioactivity	Pinese et al., (2017) [83]
Protamine, a mixture of Protamine and Melittin and Melimine	Commercial contact lens (Etafilcon-A)	Physical adsorption and covalent attachment via EDC	*P. aeruginosa*, *S. aureus*	Willcox et al., (2008) [84]
hLF1-11	Titanium surface silanized with CPTES or APTES	Peptide physical adsorption and covalent binding with CPTES or APTES	*S. sanguinis*, *L. salivarius*	Godoy-Gallardo et al., (2014) [85]
Dhvar5	Titanium and gold substrates	Physical adsorption and covalent binding with chitosan films, EDC-NHS chemistry and introduction of amino acids spacers	*S. aureus*	Costa et al., (2015) [66]
Defensin from *Anopheles gambiae* mosquitoes	Multilayer polyelectrolyte films of PEI-(PSS-PAH)_2_-(PGA-PLL)_n_	Layer-by-layer	*M. luteus*, *E. coli*	Etienne et al., (2004) [86]
Gramicidin A complexed with a non-denaturing anionic amphiphilic polysaccharide	Multilayer polyelectrolyte films of PEI and PLL	Layer-by-layer	*E. faecalis*	Guyomard et al., (2008) [87]
Ponericin G1	Silicone substrate	Layer-by-layer	*S. aureus*	Shukla et al., (2010) [88]
Magainin II	Stainless Steel	Modification of peptide with dopamine, direct grafting via catechol groups	*C. farmer*, *V. natriegens*	Cao et al., (2020) [89]
HHC-36	PU substrates	*Via* APTES-Br SAM	*E. coli*, *S. aureus*	Zhang et al. 2019 [90]
MSI-78A	Au	SAMS EG4 thiols-biotin-neutravidin-maleimide	*H. pilori*	Parreira et al. 2019 [91]
MSI-78	CaF_é_	Silica layer + maleimide	*E. coli*	Xiao et al. 2018 [92]

### 4.1. Cross-Linking of Peptides to Surfaces

**Figure 5:**
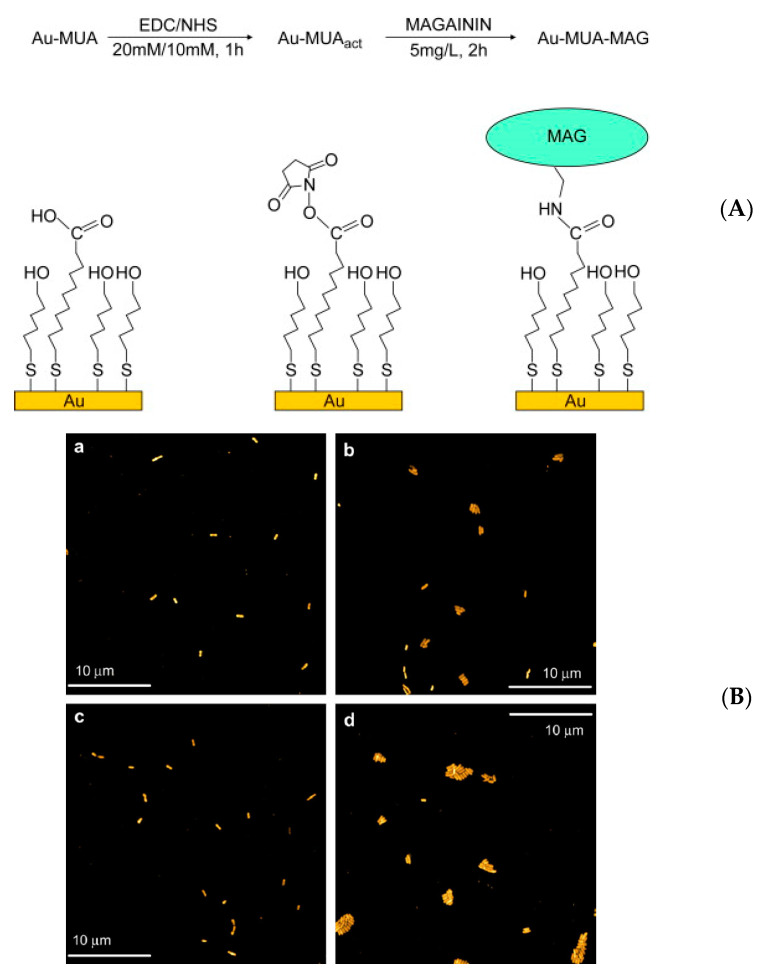
(**A**) Scheme of magainin I immobilization. Step 1: formation of SAM of MUA on gold, Au-MUA; step 2: esterification of the COOH function of MUA by NHS/EDC, Au–MUA_act_; step 3: covalent binding of magainin I, Au–MUA–MAG (**B**) AFM images of gold samples Au–MUA (**a**,**c**) and Au–MUA–MAG (**b**,**d**) obtained after 30 min and 3 h, respectively, of contact with a *Listeria ivanovii* bacterial suspension at 1.5 × 10^6^ cfu/mL at 37 °C; imaging conditions: 50 µm × 50 µm, 512 lines, 2 Hz, tapping mode. Reproduced from [71]. Copyright © 2009 Elsevier Ltd. All rights reserved.

Surface functionalization is a convenient method for covalent attachment of AMPs: a terminal function, often amine or acid, is assembled on the material surface and AMPs are attached via a cross linking agent. The case of Magainin I (MAG) immobilized on gold surfaces through Self-Assembled Monolayers (SAMs) has been studied by Humblot et al. [71] in 2009. The mechanism of action of these AMPs focuses on targeting the lipid matrix rather than the proteins present in bacteria. As shown in Figure 5, three main steps are necessary to immobilize MAG, thus a mixed SAM with 11-mercaptoundecanoic acid (MUA) was firstly elaborated. Then, through the reaction with NHS in the presence of EDC, the carboxylic acid tail groups of MUA were converted into esters. Lastly one amino moiety of MAG reacted with the ester groups via an amidation reaction to create a covalent bond between MAG and the SAM.

Adhesion tests were performed with *Listeria ivanovii* cells suspension on the resulting magainin modified surfaces. After the first 30 min of contact, it was assumed that cells could adhere but not grow within the experimental conditions used; however, IR analysis after three hours of contact showed an increase in amide bands intensity with respect to those measured after 30 min. Time-dependent adhesion was also visualized by AFM as shown in Figure 5. Additional antibacterial tests showed that more than 50% of adhered bacteria were killed, for all three types of Gram-positive bacteria tested (*L. ivanovii*, *S. aureus* and *E. faecalis*), with more than 80% in the case of *L. ivanovii*. Long-term antibacterial activity was also tested, revealing that activity persisted over six months after the first coating. With the presented results, and the fact that the concentration of MAG on the surface was low (around 1 peptide/nm^2^), this AMP makes for a possible candidate for applications in various fields.

**Figure 6:**
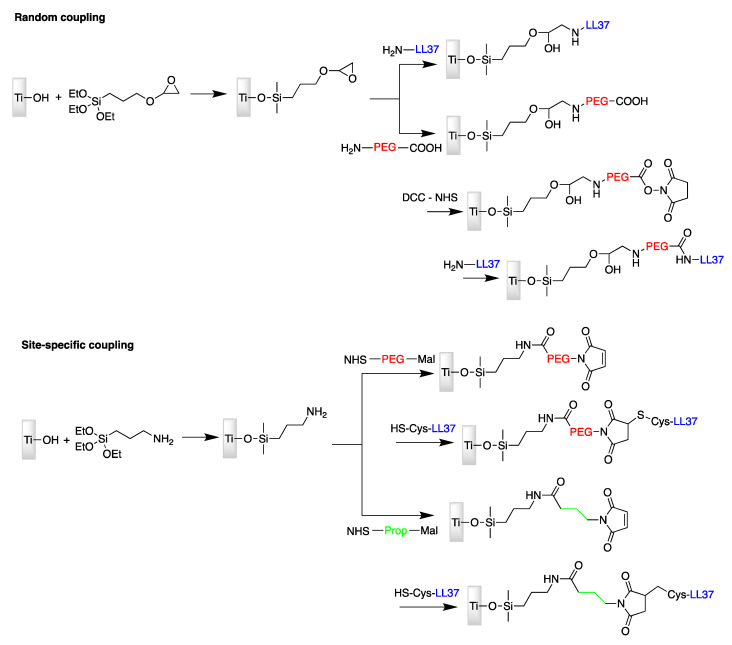
Random and site-specific strategies for coupling cathelicidin (LL-37) to titanium surface. Mal = maleimide; prop = propyl; PEG = poly(ethylene glycol).

Poly(ethylene glycol) (PEG) spacers were used to covalently attach an AMP to the surface of Titanium. Random and specific binding of peptide LL-37 has been studied in 2006 by Gabriel et al. [72]. Figure 6 displays the two strategies used in order to attach the peptide randomly via one of its six lysine residues or site-specifically from a cysteine appended to its N-terminus.

**Figure 7:**
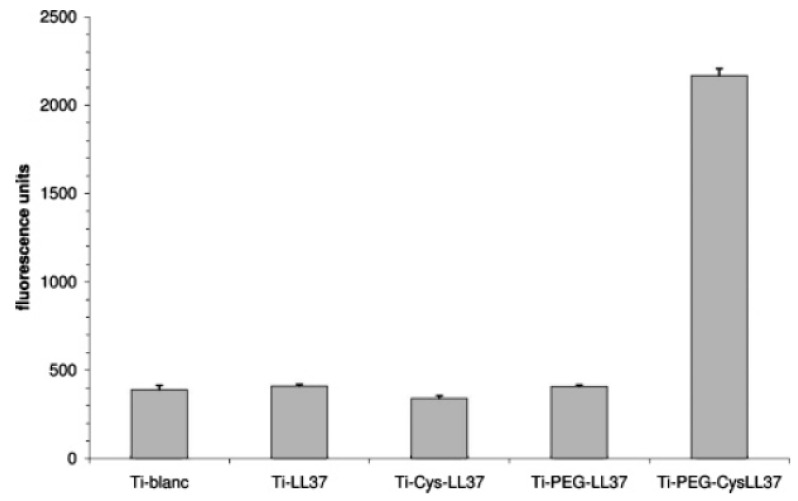
Fluorescence detection of bactericidal activity as a function of LL-37 AMP orientation and distance with respect to the surface. Reproduced with permission from [72]. Copyright 2006 American Chemical Society.

Attachment via its N-terminal function provides the immobilized peptide molecules with a parallel orientation with respect to both the surface and the incoming bacteria, which was assumed to be important for this AMP to maintain an effective antibacterial activity. To measure the amount of AMP molecules bound to the surface, a sulfo-SDTB test was used, showing that surfaces bearing oriented LL-37 molecules presents 10 times more peptides that in the random case. To measure the antibacterial activity, the authors performed killing assays against the Gram-negative bacteria *E. coli* (Figure 7). The results suggested that the bactericidal activity was not related to the relative peptide concentration on titanium surfaces, but rather to the proper orientation of the peptide as well as a sufficient distance between the AMPs and the surface.

**Figure 8:**
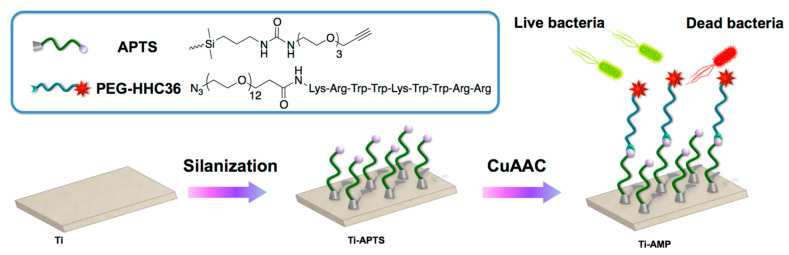
Schematic illustration of the CuAAC click chemistry on the titanium surface. Reproduced with permission from [73]. Copyright 2019 American Chemical Society.

Titanium is an interesting material, as it makes up a large part of dental implants or bone prosthesis used today. Hence, the importance of making it resistant to bacterial infections, together with other treatments in favor of bone reconstruction, osteointegration and biocompatibility for instance. In 2018, Chen et al. [73] chose to use click chemistry to produce antibacterial titanium surfaces (Figure 8). Silanization of the surface with APTS followed by coupling of the peptide HHC36 previously modified with an azido-terminated PEG chain enabled to orientate the molecule. The coupling is carried out thanks to CuAAC click chemistry on the functionalized titanium surface. This functionalization has been validated by fluorescence imaging, XPS measurements and AFM as well. In vitro antimicrobial activity has been validated with antimicrobial assays against *S. aureus* and *E. coli* (measurements of stability, cytotoxicity and fluorescence assays have been performed). The authors also managed to perform in vivo studies with six New Zealand rabbits. After one week of incubation, it seems that AMPs still display antibacterial activity which is encouraging for future developments.

**Figure 9:**
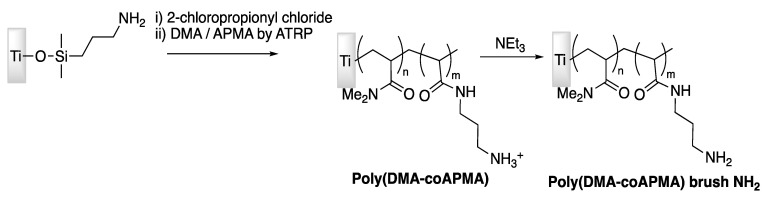
Synthetic route for copolymer brushes generation on titanium surface.

**Figure 10:**
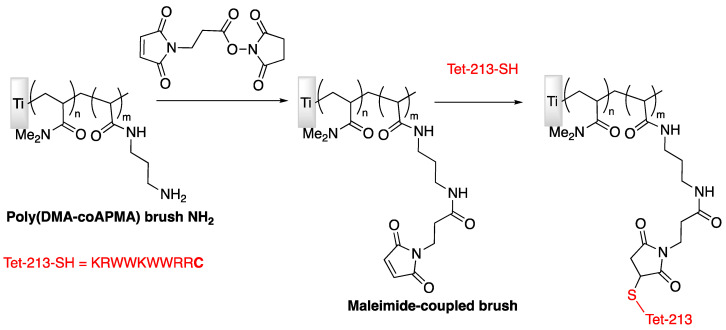
Different steps of functionalization to study the influence of brush properties of polymers on AMPs activity. Adapted with permission from [74]. Copyright 2011 American Chemical Society.

Brush properties of polymers might influence AMP immobilization and antimicrobial activity; Gao et al. [74] have then studied this particular effect in 2011 by chemical modification of titanium surface and carried out the grafting of peptide Tet-213. In this study, primary amine-functionalized copolymer brushes were generated onto titanium surfaces through surface-initiated atom transfer radical polymerization (SI-ATRP, Figure 9). After reaction of a maleimide-based heterobifunctional crosslinker, the Tet-213 modified peptide was covalently grafted via an added cysteine at the C-terminus, Figure 10.

Water contact angle measurements, XPS, ellipsometry, ATR-FTIR, and AFM were used to characterize the properties of the polymer and surfaces. The structure of the copolymer brush and the antimicrobial activity of the conjugated polymer layer were correlated. The antimicrobial activity was tested against a bacterial strain of *P. aeruginosa*. Overall, an increase in peptide surface density, grafted polymer brush density, or brush thickness, resulted in an increase of antimicrobial activity on the surface. On the other hand, the composition of the copolymers did not show a significant influence on antimicrobial activity. Through AFM, the authors verified that the brush alone was less hydrophobic than the grafted surface. Although the surfaces with higher brush density had more peptide surface density, they were less adhesive than low density brushes, which showed better antimicrobial properties. Thus, to obtain highly effective antimicrobial coatings, the optimization of graft density and peptide density are parameters of utmost importance.

The same group in 2011 (Gao et al. [75]) employed the same strategy to covalently graft various AMPs on titanium implants. In this work, N-substituted polyacrylamide brushes were covalently grafted onto surface as shown in Figure 10.

**Figure 11:**
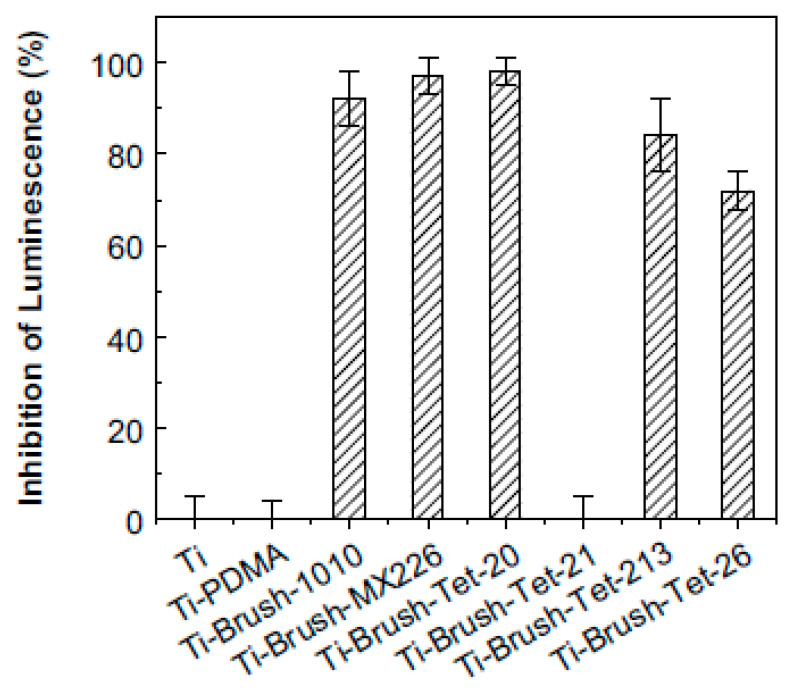
Antimicrobial activity of surface-immobilized peptides. Inhibition of luminescence of *P. Aeruginosa* upon incubation with titanium slides modified with peptide immobilized copolymer brush (surface area: 1 cm^2^); inhibition of luminescence is taken as a measure of antimicrobial activity. Ti-slides and polydimethylacrylamide (PDMA) brush coated Ti-slides (neutral brushes) were used as controls. Reproduced with permission from [75]. Copyright 2011 American Chemical Society.

The antimicrobial efficiency of the brushes was tested against a strain of *P. aeruginosa*, through a previously developed luminescence screening method. The results showed that upon immobilization of the polymer brush, the AMPs strongly retained their antimicrobial activity (Figure 11). Afterward, the the immobilization strategy giving best IL (inhibition of luminescence) value against the *P. aeruginosa* strain was then tested in vitro against Gram-negative and Gram-positive bacteria, *P. aeruginosa*, and *S. aureus*. In this case, Ti wires were used as the surface and the results confirmed the antimicrobial effect of the AMPs after immobilization. Another important aspect to test was the longevity of the antimicrobial activity of AMP-tethered polymer brushes. For this purpose, an in vivo model of implant-associated infections was developed. AMPs were proven to be highly effective in this setting, which meant that in a clinical setting (i.e., with less bacteria and greater use of antibiotics or other local type of medicine) the antimicrobial effectiveness of this AMP would be satisfactory. The topics of biological compatibility and toxicity were also studied. Results proved that AMPs conjugated polymer coating were nontoxic to mammalian cells. These AMPs, when tested on human blood, did not initiate complement activation, nor activate human platelets. Overall, the coating proved to be a relevant approach for preventing and fighting implant-associated infections.

**Figure 12:**
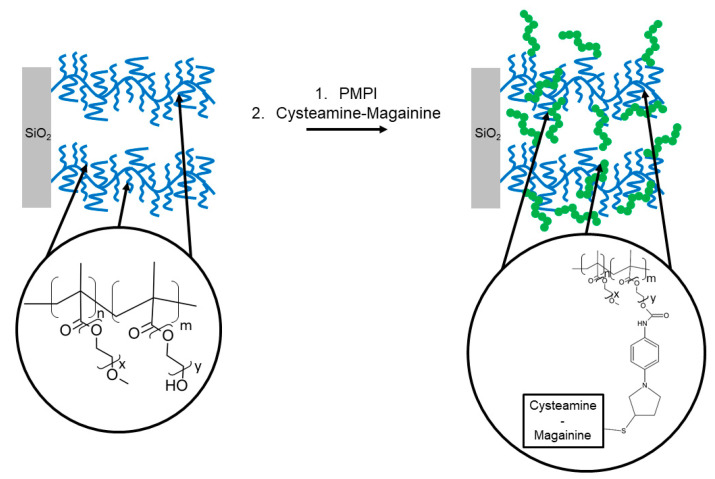
Oriented grafting of MAG-Cys derivative on poly(MOE2MA-co-HOEGMA) brushes via heterobifunctional crosslinker PMPI. Adapted with permission from [76]. Copyright 2009 American Chemical Society.

Glinel et al. [76] developed poly(MOE2MA-co-HOEGMA) brushes incorporating magainin I grafted to native oxidized silicon wafers. Modified magainin I with a C-terminal cysteine residue was tethered through an oriented chemical grafting via the heterobifunctional crosslinker PMPI (p-maleimidophenyl isocyanate) on the hydroxyl groups of the copolymer brushes (Figure 12), to preserve the activity of the peptide by maintaining mobility and accessibility. Peptide grafting was confirmed by FT-IR spectroscopy, whereas the density of grafted peptides on the brushes was determined by XPS. The antibacterial effect of Magainin I-functionalized brushes was demonstrated against two distinct strains of Gram-positive bacteria: *L. ivanovii* and *B. cereus*. The bacterial inhibition assays also demonstrated that biocidal activity of magainin I was not substantially reduced by decrease of immobilized peptide concentration.

Lim et al. [77] and Li et al. [78], studied the immobilization of engineered arginine-tryptophan rich peptides on silicone surface. In both studies, peptides were immobilized on an argon plasma-treated PDMS surface after polymerization of allyl glycidyl ether (AGE) under uv light to generate polymer brushes. Grafting was either achieved by reaction of the cysteine residue at the N-terminus of the peptide via a NH_2_-PEG-maleimide crosslinker, [77] or by reaction between the lysine residues of the peptides and the epoxide groups in the AGE brush [78]. Immobilization was confirmed by XPS. The microbiological assays done in these studies showed antibacterial activity against *E. coli*, *S. aureus*, *P. aeruginosa* in the work done by Lim and co-workers and against *E. coli*, *S. aureus* and *C. albicans* in the study carried out by Li and co-workers. Additionally, both works demonstrated anti-fouling properties of the immobilized peptides, and mostly both biocompatibility tests revealed no toxicity toward mammalian cells. Li et al. [78], successfully extended this work to the immobilization of the engineered peptides on silicone urinary catheter. Improvement of the antimicrobial activity and reduction biofilm formation were also observed.

**Figure 13:**
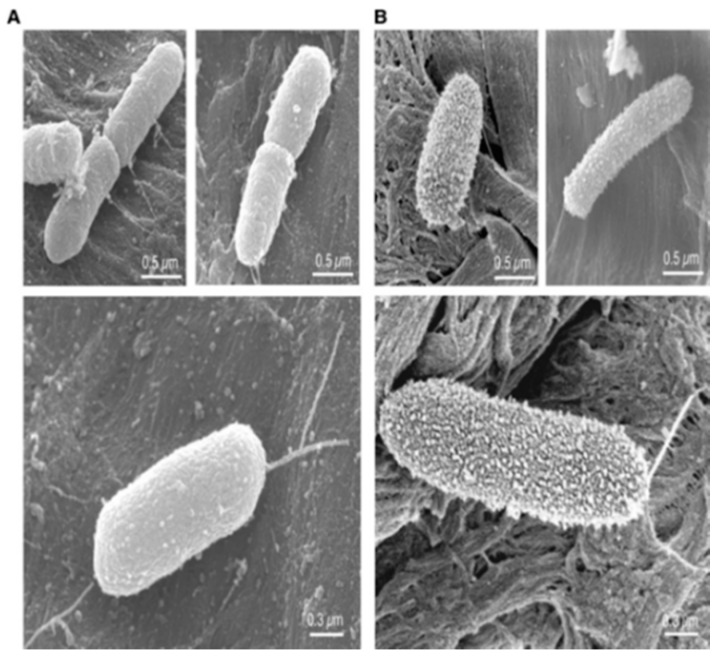
SEM images of *P. aeruginosa* in contact with cellulose membrane: (**A**) without peptide; (**B**) with an active tethered peptide. Reproduced from [80]. Copyright © 2009 Elsevier Ltd. All rights reserved.

In another field of application, developing antibacterial cellulose surfaces seems to be useful in order to prevent cosmetic or even pharmaceutical products from contamination thanks to their biocompatibility. Hilpert et al. [80] reported surface tethering of cationic peptides on cellulose support. They used among other substrates, cellulose-amino-hydroxypropyl ether (CAPE) linker chemistry to covalently immobilize several cationic peptides on the cellulose support. They employed a high-throughput screening assay strategy to identify surface bound peptides with antimicrobial activity. To this purpose, they measured luciferase production from a *P. aeruginosa* strain expressing a luciferase gene. Damages on membrane of *P. aeruginosa* caused by contact with the tethered peptides were visualized by SEM, Figure 13. Bacteria in contact with pristine cellulose membranes showed cell surfaces with smooth appearance (Figure 13A), whereas bacteria in contact with cellulose membranes with tethered peptides were characterized by the presence of blebs (Figure 13B). Structure–reactivity relationship studies on different peptides demonstrated that the antimicrobial activity was influenced by the linker and also by the peptide sequence, as the presence of cationic residues near the linker correlated with better activity and hydrophobic residues close to the N-terminus were determining factors for biological activity. SEM, ATP release, and depolarization analysis allowed Hilpert and co-workers to conclude that the perturbation of bacteria surface electrostatics must likely induce an autolytic and/or cell death mechanism. Moreover, in contrast to the soluble peptides previously studied by the same team [93], the immobilized peptides did not show any cytotoxicity.

**Figure 14:**
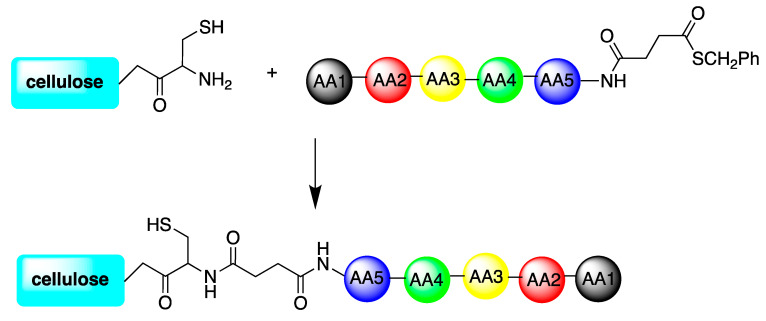
Summary of the Strategy used to covalently attach thioester peptides to cysteine-modified cellulose surfaces.

In 2020, another group [81] studied the interaction between cellulose and peptides in order to obtain antimicrobial materials. To this purpose, Sperandeo et al. synthesized a cysteine-modified cellulose conjugate to modify the surface. From another side, they synthesized two peptides (TBKKG6A and lasioglossin-III) carrying a thioester group either on the C-terminal or the N-terminal side of the molecules. Thanks to a chemical ligation reaction, peptides were grafted to cellulose as proven by FT-IR measurements so as to obtain an antimicrobial surface. The strategy is schematized in Figure 14. The antimicrobial activity was successfully evaluated against *E. coli*. The two modified AMPs prevented bacterial growth and decreased the number of viable cells. In addition, anti-bacterial activity was stronger with peptides attached via the N-terminus.

In summary, cross-linking strategies have been successfully used to graft various antimicrobial peptides to various inorganic and organic substrates. The density, the relative orientation of the peptides and the distance from the surface were highlighted as important parameters determining the overall antibacterial activity of the substrates.

### 4.2. Direct Grafting of Modified Peptides to the Surface

Through the last examples showing a possible favorable orientation of grafted peptides, modifying directly peptides in order to graft it in a one-pot fashion to the surface then appears as a good alternative way to physisorption and chemical cross linking.

**Figure 15:**
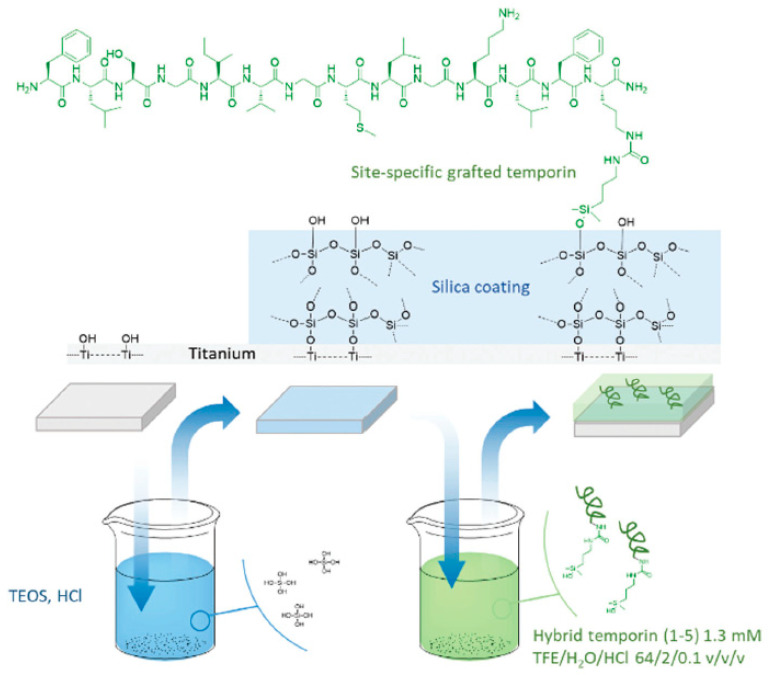
Example of site-specific grafting of hybrid temporin 1 on titanium surfaces. Temporins were modified at their C-ter end by a silanol group that enables the direct grafting onto TEOS precursor. Reproduced from [79]. Copyright © The Royal Society of Chemistry 2018.

In 2018, Masurier et al. [79], showed the possibility to graft temporin SHa via an additional chemical moiety on specific surface sites. Temporins are a family of AMPs with α-helical structure derived from frog *Pelophylax saharica* skin which main activity is exerted against the *Leishmania* genus. Temporins also show a wide spectrum of antibacterial activity against both Gram-positive and Gram-negative bacterial strains, fungi, parasites and viruses. The authors observed differences in antibacterial activity when grafted via a specific site or another. Figure 15 shows the one-step grafting strategy of temporin-SHa carrying a dimethylhydroxysilane group to titanium surfaces, in the attempt to directly react the modified surface with the hybrid peptide. However, the formed Si-O-Ti bonds can be easily hydrolyzed, and therefore the grafting density reduced after incubation. However, if the titanium surface was previously coated with silica, Si-O-Si bonds will form instead of Si-O-Ti. These new bonds, although still hydrolyzable, are much stronger than Si-O-Ti. Therefore, the titanium substrate was coated with silica prior to peptide immobilization. XPS was used to confirm the successful grafting and measure the thickness of the silica coating and the surface density. Independently of the orientation of the grafted molecules, the determined molecular surface density and the number of grafted molecules were similar. They also observed a better interaction with the bacterial membrane and so a higher antibacterial activity when the dimethylhydroxysilane function is introduced in the middle of the sequence, rather than at the N-terminus or the C-terminus.

**Figure 16:**
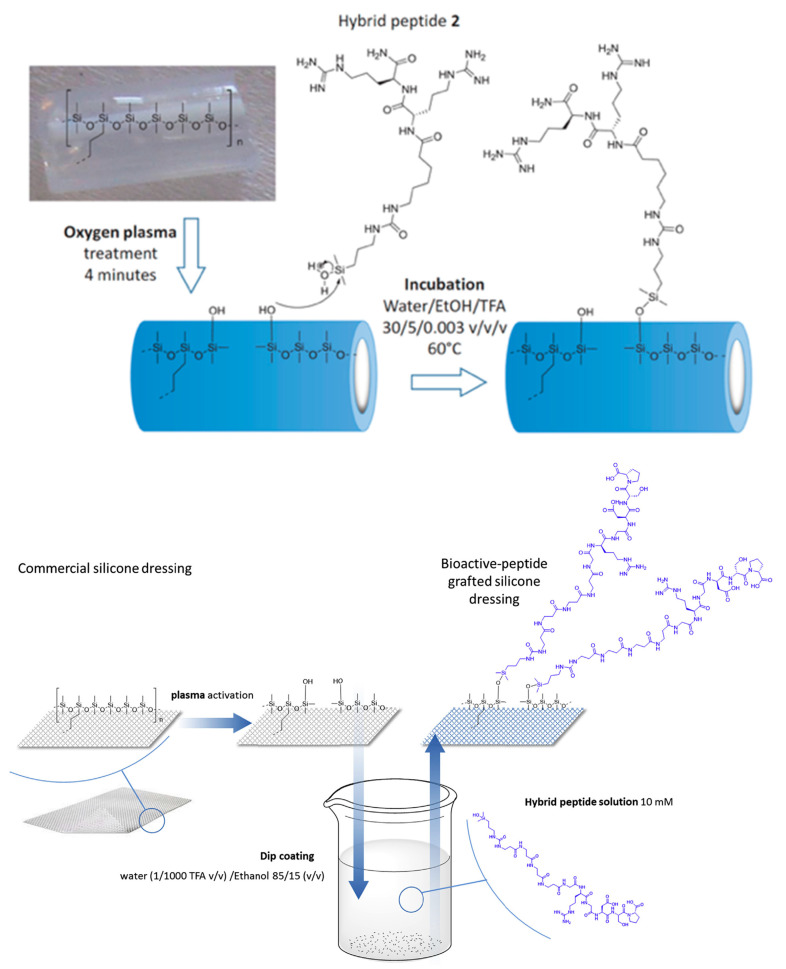

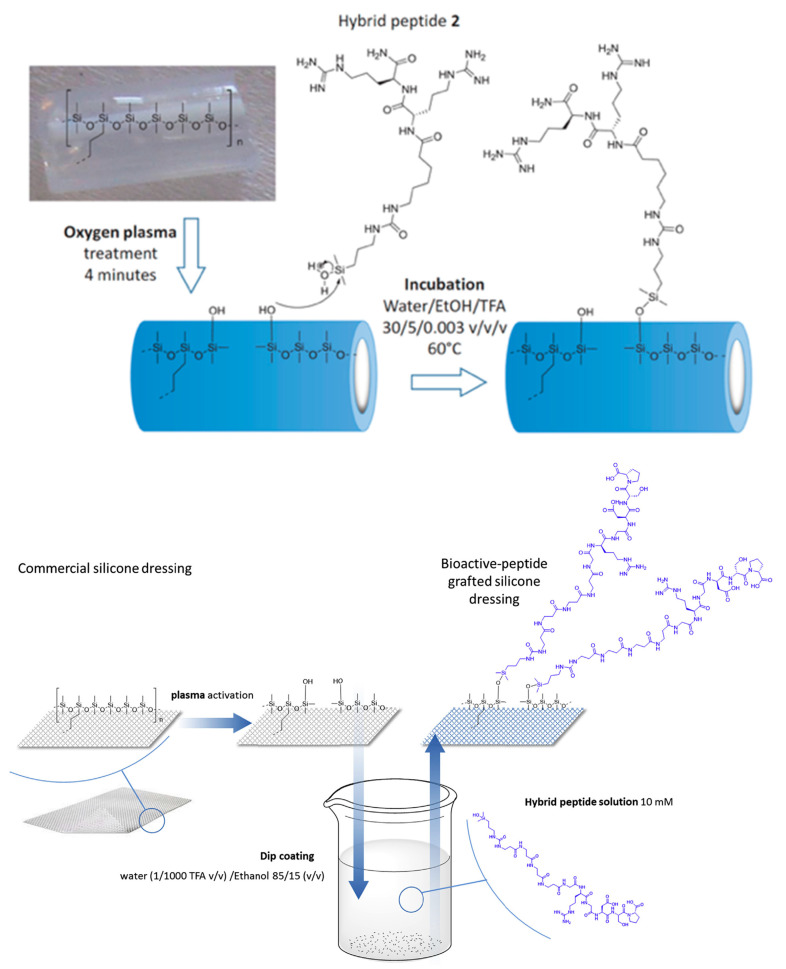
Grafting of AMP on activated silicone catheter (**top**) and on activated silicone dressing (**bottom**). For both strategies, the commercial silicone is activated by plasma before direct grafting of the modified peptide by a silanol group. Reproduced from [82,83]. Copyright © 2016 WILEY-VCH Verlag GmbH & Co. KGaA, Weinheim. Copyright © 2017 Elsevier Ltd. All rights reserved.

In another examples, and following the same strategy, Pinese et al. studied silicone surfaces mimicking silicone catheters [82] and silicone dressings [83]. The authors investigated a strategy to make peptides grafting easier and more suitable for the fabrication of medical devices. Additionally, they tried to prevent peptide release in the biological fluids of the wound in order to maintain a good biological effect. To achieve this, they proposed to use hybrid dimethylhydroxysilyl peptides to covalently bind the peptides through Si-O-Si bonds to a plasma-activated silicone surface. The grafting process is similar for both works. First, the silicone surface was activated by oxygen plasma treatment to generate active silanol groups. Then, to properly achieve the grafting, the silicone catheter was incubated in a solution containing the hybrid peptide (Figure 16 top), whereas for the silicone dressing the bioactive peptide was grafted by dip-coating (Figure 16 bottom), which allows for treatment of devices on a larger scale.

This grafting process via covalent adsorption directly on the silicone surface led to a relatively low density of active peptides (~0.1 peptide/nm^2^ of silicone on the catheter and ~0.4 peptide/nm^2^ of silicone on the dressing). As a comparison, adsorption of antimicrobial peptides on gold surface functionalized by SAM was found to be ~1 peptide/nm^2^ [94], and also the density of Lasio II antimicrobial peptide immobilized on a polymer brushes layer grafted on silicone catheters was found to be 21 peptides/nm^2^ [95]. Nevertheless, the relatively low density observed by Pinese and co-workers, partnered with the mode of association of the bioactive peptide with the substrate, was not detrimental to the bioactivity of the surface, showing on the contrary very good bactericidal properties together with very low cytotoxicity.

**Figure 17:**
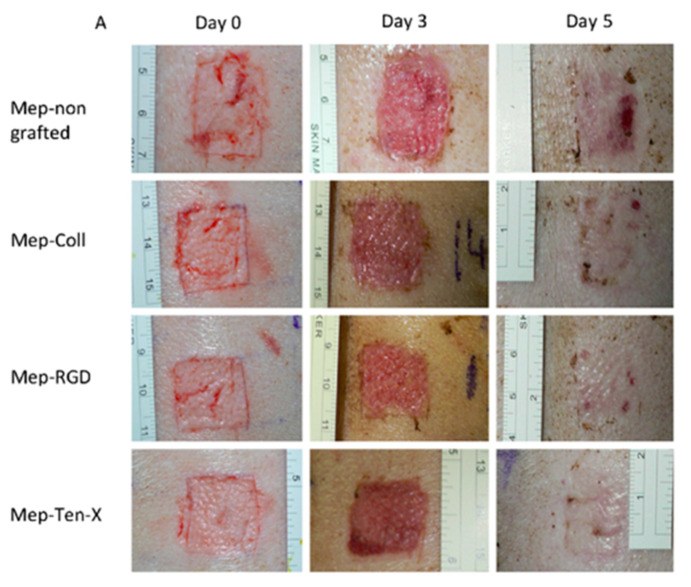

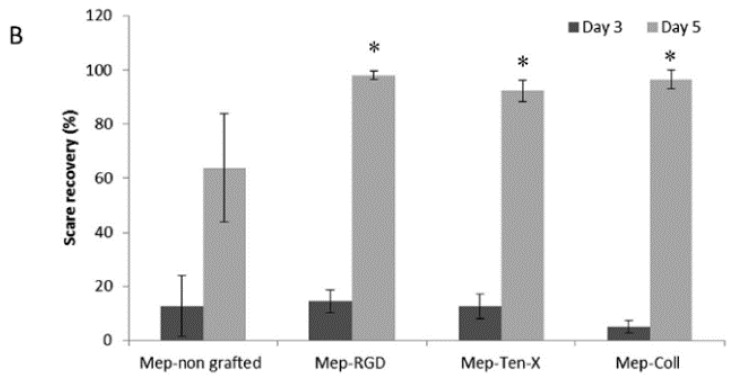
In vivo study. (**A**) Pictures of wounds inflicted on the back of a pig (at days 0, 3 and 5); (**B**) percentage of recovery of the scars (peptide-treated vs. non-treated dressings). Reproduced from [83]. Copyright © 2017 Elsevier Ltd. All rights reserved. Statistically significant differences vs. control samples are indicated with an * (*p* < 0.05).

In their work on grafting of antibacterial peptides on silicone catheters [82], Pinese et al. tested the antibacterial activities on the three most commonly found bacteria in contaminated medical devices: *E. coli*, *P. aeruginosa* and *S. aureus*. They demonstrated a 75% reduction in the number of bacteria found on the peptide-grafted surfaces after 24 h of incubation in a solution containing a known number of bacteria, by comparison with a non-grafted silicone surface. These results are satisfactory, and they further demonstrated that the peptide retains its antibacterial activity when covalently grafted. Additionally, the peptide-grafted silicone surfaces demonstrated strong anti-fouling properties on the adhesion of *S. aureus* as they were more effective than commercial “silver-coated” catheters, which are associated with a potential toxicity upon accumulation of silver in tissues and the development of silver-resistant bacteria [96,97]. The long-term efficiency of these antibacterial peptide-grafted silicone catheters is also higher than the commercial “silver-coated” materials. The work of the same group on bioactive peptides grafted on silicone dressings [83] aims to bring bioactivity to the dressing through the immobilization of three bioactive hybrid peptides. In vitro studies showed that peptide-grafted silicone dressings improved cell adhesion and proliferation in wound zone, favoring wound healing. Additionally, these dressings promoted extracellular matrix synthesis by enhancing collagen and fibronectin synthesis. Wounds inflicted on the back of a pig confirmed the in vitro studies, showing that grafted dressings can improve healing by allowing a significantly increased scar recovery (Figure 17A,B).

One major challenge arises when it comes to keep AMPs antibacterial activity once immobilized and limit the toxicity potentially caused by their release. Several works done on silicone substrates have shown that it is possible to keep the antibacterial and more generally the biocidal effect [83] after grafting, which is very encouraging with respect to toxicity. For this reason, strong immobilization on silicone surfaces was shown to be the preferred method. Grafting through the use of a linker or polymeric brushes have been reported in several works, however an easier model was studied by Pinese et al., in which the bioactive peptide is directly grafted to the reactive silanol groups of plasma-activated silicone surface. Moreover, several studies showed that it is possible to achieve strong antibacterial activity with relatively low quantities of immobilized peptide on the surface [76,82,83].

In summary, direct grafting of modified peptides has been successfully achieved on several kind of surfaces from metal ones to polymers. The grafting has revealed to be simple and efficient; low concentration of active molecules enables very high antibacterial activities compared to classical multistep grafting strategies. This route seems to be a promising one in term of cost efficiency due to the low density of active molecules required to achieve high bactericidal grafting.

### 4.3. Comparison between Covalent Attachment and Physical Adsorption

**Figure 18:**
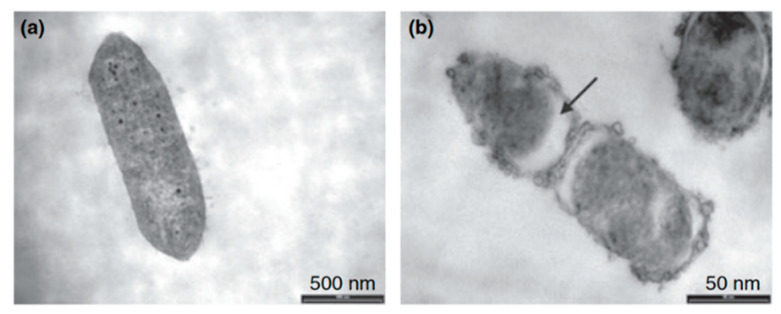
Transmission electron microscopy (**a**) *Pseudomonas aeruginosa* strain 6294; (**b**) *P. aeruginosa* Scheme 6294. exposed to melimine. Reproduced from [84]. © 2008 Willcox et al. J. Appl. Microbiol. © 2008 The Society for Applied Microbiology.

In order to compare covalent attachment and physical adsorption, Willcox et al. [84] reported the use of cationic peptides to prevent microbial colonization of commercialized Etafilcon A, a contact lenses material made of hydroxyethyl methacrylate/methacrylic acid (HEMA/MAA) copolymer. Protamine, a mixture of protamine and melittin and a synthetic peptide melimine were the AMPs used for this purpose. They studied two modes of attachment: physical adsorption and covalent attachment via 1-ethyl-3-(3-dimethylaminopropyl) carbodiimide (EDC) coupling reaction for melimine. This AMP retained the combined anti-microbial activity of protamine and melittin against both *P. aeruginosa* and *S. aureus* by reducing bacterial adhesion to the polymeric surface, while being less toxic to red blood cells. Willcox and co-workers also demonstrated that melimine retains its activity while covalently attached to the polymer. The peptide showed a concentration-dependent activity to reduce bacterial colonization (in over 80%) with good broad-spectrum efficacy at 500 g per lens. Covalent coupling showed greater activity, as only 20 g per lens reduced bacterial adhesion in 70%. Moreover, exposure of bacteria to melimine at MIC concentration for 30 min at 37 °C resulted in structural changes of the bacterial cells. As shown in Figure 18, *Pseudomonas aeruginosa* cells show membrane bleeding at the cell surface, disintegration of the membrane, condensation of the contents (mainly DNA) and detachment of the cell membrane from the cell wall (black arrow in Figure 18b). Moreover, no melimine-induced resistance was observed after repeated exposure of bacteria to sub-inhibitory concentrations of the peptide, making this peptide a viable option to prevent possible infections via contact lenses. Above all, covalent attachment displays more efficiency than physical adsorption.

**Figure 19:**
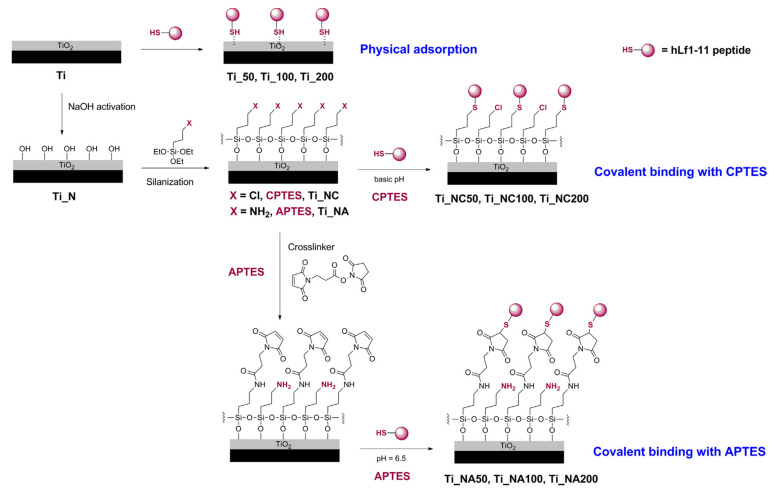
Functionalization strategies to anchor the hLf1-11 peptide to titanium: physical adsorption and covalent binding with CPTES or APTES. Adapted from [85]. Copyright © 2014 Acta Materialia Inc. Published by Elsevier Ltd. All rights reserved.

Godoy-Gallardo et al. [85] studied the antibacterial activity of hLf1-11, inspired from lactoferrin, by functionalizing titanium surface by silanization with CPTES (3-chloropropyltriethoxysilane) or APTES (3-aminopropyltriethoxysilane), the covalent binding is confirmed by XPS, or physisorption, Figure 19. The antimicrobial activity of the peptide was tested against *S. sanguinis* and *L. salivarius* (useful for dental applications).

**Figure 20:**
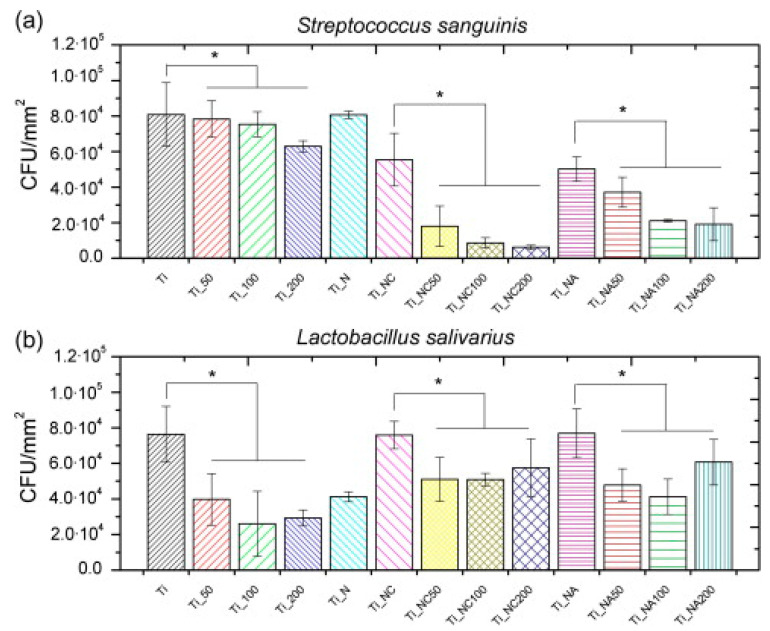
Bacterial adhesion of *S. sanguinis* (**a**) and *L. salivarius* (**b**) to titanium samples after 2 h of incubation at 37 °C. Statistically significant differences vs. control samples are indicated with an * (*p* < 0.05). Reproduced from [85]. Copyright © 2014 Acta Materialia Inc. Published by Elsevier Ltd. All rights reserved.

They prepared different titanium surfaces: Ti (smooth titanium), Ti_N (titanium pretreated with NaOH), Ti_NA (titanium pretreated with NaOH and silanized with APTES) and Ti_NC (titanium pretreated with NaOH and silanized with CPTES). Samples were functionalized by physisorption at different concentrations of Lf1-11 peptide: Ti_50, Ti_100, Ti_200. Finally other substrates were functionalized by covalent binding: Ti_NA50, Ti_NA100, Ti_NA200 and Ti_NC50, Ti_NC100, Ti_NC200. Results showed an inhibition of biofilm formation and development for both bacteria strains. Indeed, with a CFU/mm^2^ enumeration with control Titanium surfaces, the effect of AMPs is widely shown especially for covalent bindings, Figure 20. Indeed, the bacterial adhesion of *S. sanguinis* is similar for the control and the case of physical adsorption with a value around 8.0 × 10^4^ CFU/mm^2^. Whereas for covalent binding with CPTES the value is around 1.0 × 10^4^ CFU/mm^2^ and with APTES around 3.0 × 10^4^ CFU/mm^2^. Again, covalent binding is more suitable to achieve a better antibacterial activity.

**Figure 21:**
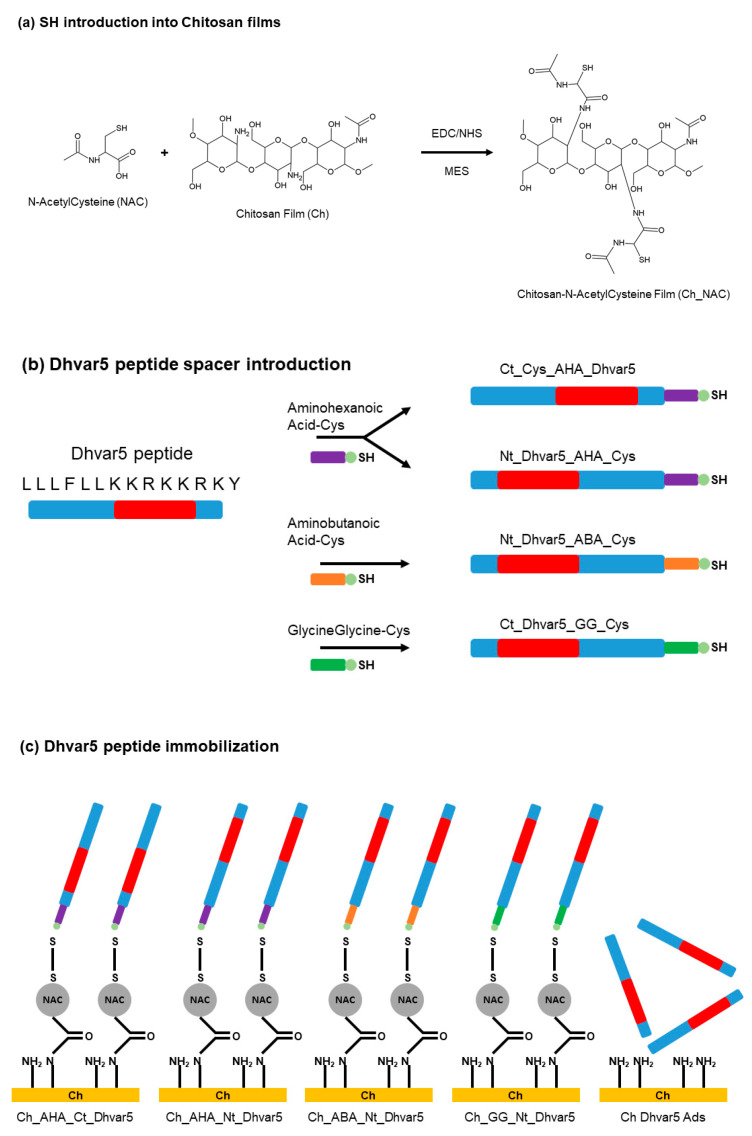
(**a**) Chitosan modification with N-acetyl cysteine (NAC); (**b**) Dhvar5 peptide derived in Table 2 peptides by disulfide bridge formation (a control surface, Ch Dhvar5 ads, was used where peptide was only physisorbed, not covalently bound). (**c**) Dhvar5 peptide immobilization strategy. Reproduced from [98]. Copyright © 2015 Elsevier Ltd. All rights reserved.

Finally, a study of 2015 by Costa et al. [98], showed also that covalent immobilization of AMPs is more efficient than physical adsorption. Indeed, the ability to control the orientation of the peptide and its distance to the surface is crucial. Peptide Dhvar5 was covalently immobilized on titanium and gold substrates. After deposition of chitosan films after their functionalization with N-acetyl cysteine (Figure 21a), the peptide was linked to the surface via a disulfide bridge with its terminal cysteine (Figure 21c). This process is confirmed thanks to FT-RAIRS, Ellipsometry and water contact angle measurements. Additionally, physical adsorption is achieved thanks to hydrophobic end of the biomolecule. This chemistry is summed up on Figure 21. Antibacterial tests against *Staphylococcus aureus* proved the ability of covalently bound AMPs to decrease the colonization whereas for physical adsorption, there is no effect and even worse it can sometimes facilitate bacterial adhesion. Moreover, the introduction of spacers (Figure 21b), showed that the best antibacterial activity is obtained for the longest possible distance between the surface and the peptide.

To summarize this section, these three studies show for three different materials (copolymer, titania and gold) the advantage of using covalent linked AMPs rather than physically adsorbed peptides. Another approach exists, layer-by-layer, and will be studied in the last part of the review.

### 4.4. Layer-by-Layer Approach

The Layer-by-Layer (LbL) technique is based on the alternating deposition of polycations and polyanions onto a surface. LbL represents a simple and robust method to coat various surfaces and has been explored in several studies for the immobilization of AMPs on the surface of materials. Experimentally, peptides are directly incorporated in the polyelectrolyte multilayers (PEM) by the LbL method.

**Figure 22:**
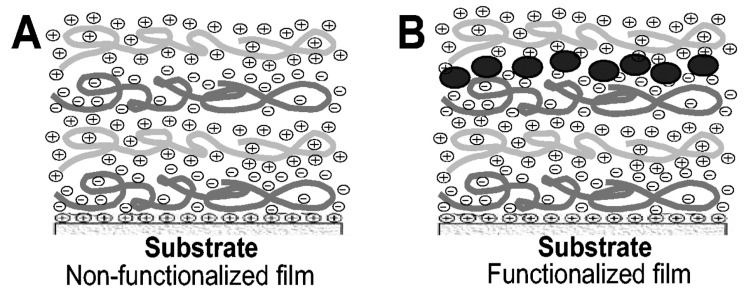
Scheme of polyelectrolyte multilayer film (**A**) and defensin insertion (**B**). Adsorption is obtained by adding a layer of the opposite charge; the peptide is embedded under another polyelectrolyte layer. Reproduced from [86]. Copyright © 2004 American Society for Microbiology.

Etienne et al. [86], have successfully functionalized PEM with defensin from *Anopheles gambiae* mosquitoes. They studied the influence of the number of peptide layers on bioactivity and observed a 98% inhibition of *Escherichia coli* growth when 10 layers of antimicrobial peptides were embedded in the polyelectrolyte film architecture. The architecture of the film is shown on Figure 22. Additionally, good biocompatibility was demonstrated as some of the multilayer films revealed to be stable for more than two weeks in presence of body fluids. However, as defensin is positively charged (pI = 10) at the working pH of this study (pH = 6.5–7), this approach is restricted to highly-charged soluble AMPs that interact through electrostatic interactions with the polyelectrolyte chains constituting the PEM. Moreover, strong electrostatic interactions between the peptide and the polyelectrolytes may lead to denaturation of the peptide, therefore limiting its antibacterial activity.

**Figure 23:**
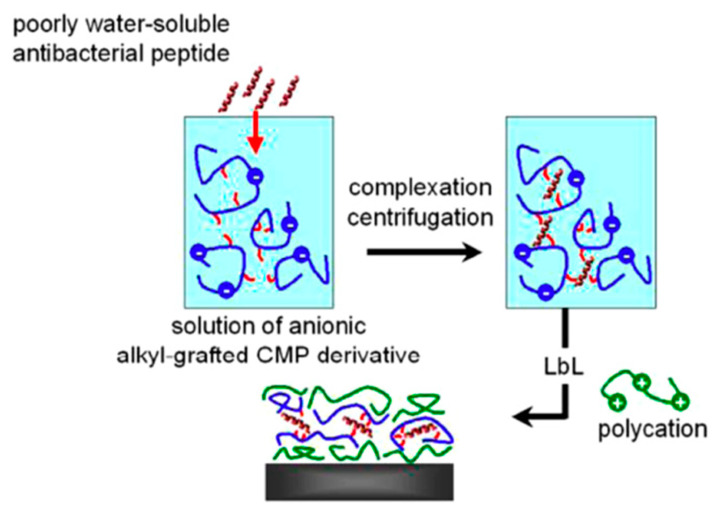
Process used for immobilization of Gramicidin A. Reproduced from [87]. Copyright © 2008 WILEY-VCH Verlag GmbH & Co. KGaA, Weinheim.

Guyomard et al. [87], proposed an alternative approach to insert AMPs into polyelectrolyte films. The strategy is based on the insertion of Gramicidin A (Gram A), a hydrophobic antibacterial peptide, into a polyelectrolyte multilayer to elaborate biocidal films following the process described in Figure 23. Firstly, hydrophobic Gram A molecules were trapped by amphiphilic alkyl-grafted Carboxymethylpullulans (CMP), leading to their solubilization. Then, the resulting anionic “complex” was assembled by a LbL process with cationic poly(L-Lysine) (PLL) to form the peptide-functionalized films. These resulting films were shown to contain hydrophobic nanodomains in which gramicidin A was entrapped. The antibacterial activity of the resulting functionalized films was demonstrated against Gram-positive bacteria, *Enterococcus faecalis*. The authors also showed that biocidal activity proceeded through a double mechanism of contact between bacteria and film surface, and slow release of the peptide into the solution surrounding the films. In addition, rinsing of multilayers did not completely remove the peptide, which confirmed that gramicidin A was slowly released, therefore suggesting a long-term preservation of the biocidal activity of the film surface.

**Figure 24:**
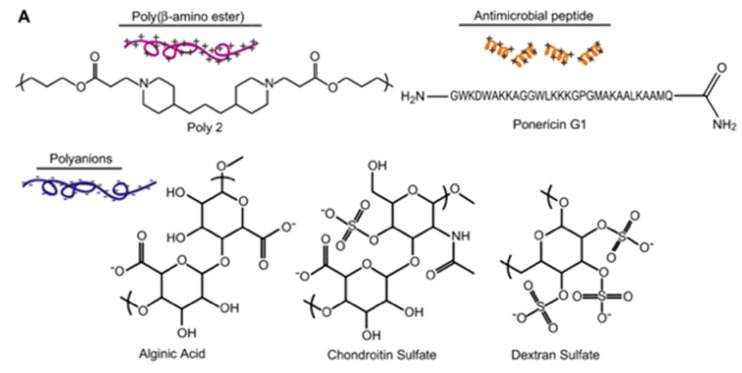

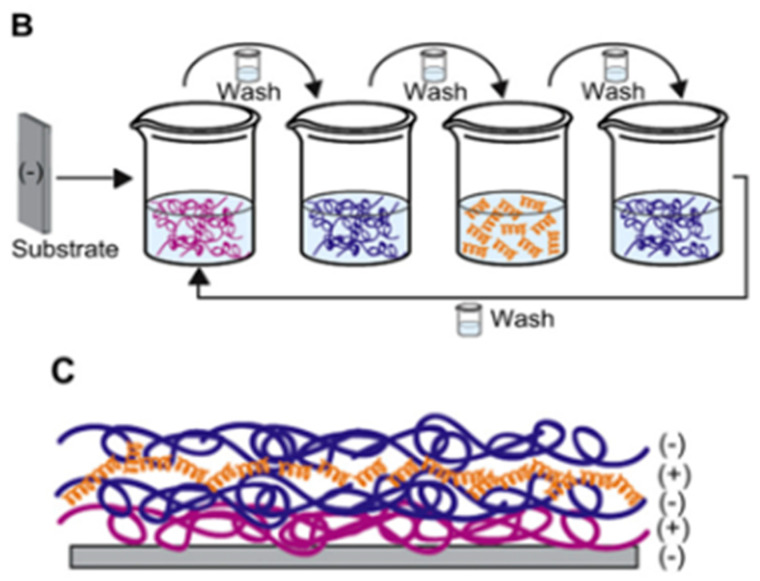
Process used for the immobilization of ponericin G1 embedded with the LbL structure (**A**) structures of all polyelectrolytes used in the LbL process. (**B**) schematic protocol of LbL elaboration and (**C**) Schematic expected structure of the final LbL film. Reproduced from [88]. Copyright © 2009 Elsevier Ltd. All rights reserved.

Finally, Shukla et al. [88], proposed to control the release of AMPs incorporated in PEM by using hydrolytically degradable multilayer films. They worked on local delivery of ponericin G1, an AMP exhibiting a net positive charge at physiological pH and active against *S. aureus*, as therapeutic agent for wound healing process. Ponericin G1 was embedded into a LbL assembled polyelectrolyte multilayer film based on a hydrolytically degradable cationic poly(β-aminoester) and an anionic non-cytotoxic polyanion such as alginic acid, deposited on a silicone surface. The process is described in Figure 24.

Several film architectures were studied in order to obtain various peptide loadings and led to varying release profiles in order to control the long-term activity of the films. The resulting films were able to inhibit *S. aureus* attachment for over 10 days, as degradation of the poly(β-aminoester) leads to slow release of the peptide. These observations indicated that film-releasing peptides did not suffer any loss of antibacterial activity. In addition to prevent biofilm formation, all films were found to be biocompatible with the wound healing cells.

In several works the incorporation of AMPs in PEM through a LbL process was used to immobilize bioactive peptides. As peptides are generally charged, electrostatic interactions between AMP and the polyelectrolytes represent an obstacle for the stability of these polyelectrolyte multilayers films incorporating the AMP. Nevertheless, trapping of AMP in hydrophobic nanodomains in these films was shown to be a good alternative to ensure a better stability of these films, allowing to maintain the biocidal activity. Release of AMP from PEM films can in some cases, for the delivery of a wound healing peptide for instance, be desired and this release can be controlled by engineering of the film architecture.

## 5. Conclusions and Future Prospects

Antimicrobial peptides naturally existing in the animal and plant kingdoms prompted the development of natural and bio-inspired synthetic peptides active against biofilms. The potential of AMPs as an anti-infective agents has been noted, leading for many of them to clinical trials in order to get approval as marketed drugs [99]. Despite multiple efforts, issues such as production cost, reduced efficiency and more often possible toxicity still stand in the way of such approval, and despite the huge amount of successful studies present in the literature, no synthetic AMP and very few natural AMPs have obtained FDA approval [100,101]. Notwithstanding promising research and on-going clinical trials and, despite still in development, positive prospects for AMP use are in sight, especially concerning combination therapies. Indeed, synergetic effect of peptides coupled with other therapeutic agents such as antibiotics or essential oils have shown great potential in increasing efficacy of treatment [102]. Furthermore, AMPs hold the crucial advantage of overcoming low susceptibility of biofilm to antimicrobial agents, which suggests that their killing efficiency is promising, albeit still far from topping that of current antibiotic treatments.

This review has highlighted the wide range of antimicrobial peptides, along with the various immobilization strategies developed to date. Indeed, these biocidal-functionalized surfaces are able to target a broad spectrum of both Gram-positive and Gram-negative bacteria, together with viruses, parasites and algae for some more specific AMPs. As could be concluded from the numerous works described, covalent-based approaches are more advantageous, in addition to represent a crucial role in antimicrobial activity. Importance of the selected immobilization technique is highly emphasized in the stated literature, in some cases dominating over factors like concentration of bound peptide [63]^,^ even via peptide engineering either by addition of a specific amino acid fragment or anchoring moiety or simply by designing totally new synthetic AMP with desired architecture and specificity [103,104]. Decades of research have led to a better understanding of the complex mechanisms of action of AMPs, however additional progress is certainly necessary to overcome current challenges and sustainably commercialize AMP-based antimicrobial coatings for use against biofilm.

## Figures and Tables

**Table 1:** AMPs in clinical trials (phase I-III) and preclinical trials (2019). Data collected from ref. [53,55,59,60,61,62].

AMP	AMP Source	Target ^a^	Phase	Company
EA-230	hCG derivative	Sepsis and renal failure	II	Exponential Biotherapies
		protection		
CZEN-002	α-MSH derivative	Anti-fungal	II	Zengen
XMP-629	BPI derivative	Impetigo and acne rosacea	III	Xoma Ltd.
Neuprex(rBPI21)	BPR derivative	Pediatric meningococcemia	III	Xoma Ltd.
Delmitide(RDP58)	HLA class I derivative	Inflammatory bowel disease	II	Genzyme
Ghrelin	Endogenous HDP	Chronic respiratory failure	II	University of Miyazaki; Papworth Hospital
NVB-302	Lantibiotic	*C. difficile*	I	Novacta
hLF1-11	Lactoferricin derivative	MRSA, *K. pneumoniae*, *L. monocytogenes*	I/II	AM-Pharma
Wap-8294A2 (Lotilibcin)	*Lysobactor* spp.	G(+) bacteria(VRE, MRSA)	I/II	aRigen
DPK-060	Kininogen derivative	Acute external otitis	II	ProMore Pharma
PXL01	Lactoferrin analog	Postsurgical adhesions	III	ProMore Pharma
PAC113	Histatin 5 analog	Oral candidiasis	II	Pacgen Biopharmaceuticals
POL7080	Protegrin analog	*P. aeruginosa* *K. pneumoniae*	III	Polyphor Ltd.
OP-145	LL-37 derivative	Chronic middle ear infection	II	Dr. Reddy’s Research
LL-37	Human cathelicidin	Leg ulcer	II	ProMore Pharma
Novexatin (NP213)	Cyclic cationic peptide	Fungal nail infection	II	Novabiotics
Iseganan (IB-367)	Protegrin analog	Pneumonia, stomatitis	III	IntraBiotics Pharmaceuticals
Pexiganan (MSI-78)	Magainin analog	Diabetic foot ulcers	III	Dipexium Pharmaceuticals
Omiganan (CLS001)	Indolicidin derivative	Rosacea	III	Cutanea Life Sciences
Surotomycin	Cyclic lipopeptide	*C. difficile* (diarrhea)	III	Cubist Pharmaceuticals/Merck
Ramoplanin (NTI-851)	*Actinoplanes* spp.	G(+) (VRE, *C. difficile*)	III	Nano-therapeutics
Friulimicin B	Cyclic lipopeptide	Pneumonia, MRSA	I	MerLion Pharmaceuticals
MU1140	Lantibiotic	G(+) bacteria (MRSA, *C. difficile*)	P	Oragenics
HB1275	Lipopeptide	Fungal skin infections	P	Helix Biomedix
HB1345	Lipopeptide	Skin infections, acne	P	Helix Biomedix
Arenicin (AP139)	*Arenicola marina*	G(−) bacteria, UTI	P	Adenium Biotech
AP114	Arenicin analog	*C. difficile*	P	Adenium Biotech
AP138	Arenicin analog	MRSA	P	Adenium Biotech
Novamycin (NP339)	Poly-arginine cationic peptide	Fungal infections	P	Novabiotics
Avidocin and Purocin	Modified bacteriocin	G(−) bacteria	P	Pylum Biosciences

^a^ G(+), Gram positive; G(−), Gram negative; MRSA, methicillin-resistant *S. aureus*; VRE, vancomycin-resistant *Enterococci;* UTI, urinary tract infection; P, preclinical.

## List of Abbreviations

AA	Amino Acid
AFM	Atomic Force Microscopy
AMP	AntiMicrobial Peptide
APD	Antimicrobial Peptide Database
APTES	3-Aminopropyltriethoxysilane
ATP	Adenosine triphosphate
ATR-IR	Attenuated Total Reflection-Infra Red
CAPE	Cellulose-amino-hydroxypropyl ether
CFU	Colony-Forming Unit
CMP	Carboxymethylpullalans
CPTES	3-Chloropropyltriethoxysilane
EDC	1-Ethyl-3-(3-dimethylaminopropyl) carbodiimide
EPS	Extra Cellular Polymeric substance
FDA	Food and Drug Administration
HEMA	hydroxyethyl methacrylate
LbL	Layer-by-Layer
IL	Inhibition of Luminescence
MAA	Methacrylate Acid
MAG	Magainin
MIC	Minimal Inhibition Concentration
MUA	11-mercaptoundecanoic acid
NHS	N-Hydroxysuccinimide
NMR	Nuclear Magnetic resonance
PDMA	Polydimethylacrylamide
PDMS	Polydimethylsiloxane
PEG	Poly(Ethylene Glycol)
PEM	Polyelectrolyte multilayers
PLL	Poly-L-ysine
PM-RAIRS	Polarisation Modulation Reflection Absorption Infrared Spectroscopy
PMPI	p-maleimidophenyl isocyanate
QS	Quorum Sensing
SAM	Self-Assembled Monolayer
SEM	Scanning Electron Microscopy
SI-ATRP	Surface-Initiated Atom Transfer Radical Polymerization
XPS	X-Ray Photoemission Spectroscopy
